# Antenatal Corticosteroids and Bronchopulmonary Dysplasia in Very Preterm Infants

**DOI:** 10.1001/jamanetworkopen.2025.45606

**Published:** 2025-11-26

**Authors:** Liang Gao, Zhi Zheng, Xin-Zhu Lin, Wei Shen

**Affiliations:** 1 Department of Neonatology, Women and Children’s Hospital, School of Medicine, Xiamen University, Xiamen, Chinas; 2 Department of Pediatrics, Women and Children’s Hospital, School of Medicine, Xiamen University, Xiamen, China

## Abstract

**Importance:**

The impact of antenatal corticosteroids (ACS) on bronchopulmonary dysplasia (BPD) in very preterm infants remains controversial, with limited evidence on causal mediation pathways.

**Objective:**

To evaluate the association between ACS and BPD in very preterm infants and assess whether respiratory distress syndrome (RDS) and invasive mechanical ventilation (IMV) have mediating roles.

**Design, Setting, and Participants:**

This prospective multicenter cohort study included preterm infants from 28 tertiary centers in China between September 1, 2019, and December 31, 2020. Inclusion criteria were gestational age (GA) less than 30 weeks, admission to the neonatal intensive care unit with 24 hours of birth, and neonatal hospitalization for more than 2 weeks. Analysis was done from April 1 to May 1, 2025.

**Exposure:**

Complete or incomplete ACS courses (vs no ACS).

**Main Outcomes and Measures:**

The primary outcome was moderate-to-severe BPD (using National Institute of Child Health and Human Development 2001 criteria) assessed at corrected GA of 36 weeks. Secondary outcomes were severe RDS (grade 3-4) and IMV duration. Regression models adjusted for demographic, pregnancy, and birth characteristics.

**Results:**

A total of 1097 preterm infants were enrolled, with median gestational age of 28.71 weeks (IQR, 27.71-29.29 weeks), median birth weight of 1150 g (IQR, 1000-1310 g), and median IMV duration of 2.0 days (IQR, 0.0-7.0 days). Of 1075 infants with available sex data, 599 (56%) were males. A total of 1069 infants had known ACS data; ACS were given in 832 cases (78%), with 518 (48%) receiving complete courses. Moderate-to-severe BPD occurred in 309 of 1097 infants (28%) and severe RDS in 237 of 1085 (22%). Complete ACS courses showed negative associations with risk of moderate-to-severe BPD (adjusted risk ratio [ARR], 0.68; 95% CI, 0.55-0.84), severe RDS (ARR, 0.67; 95% CI, 0.51-0.88), and IMV duration (β, −2.003; 95% CI, −3.391 to −0.614). Significant associations with lower BPD risk were observed in infants with GA of 28 weeks to 28 weeks 6 days (ARR, 0.47; 95% CI, 0.29-0.74), singletons (ARR, 0.67; 95% CI, 0.50-0.88), and vaginal deliveries (ARR, 0.62; 95% CI, 0.46-0.83). Mediation analysis suggested that ACS was associated with reduced risk of BPD (β, −0.050; 95% CI, −0.081 to −0.017) through direct (β, −0.031; 95% CI, −0.061 to −0.001) and indirect (β, −0.019; 95% CI, −0.032 to −0.007) effects, with the latter comprising both single and serial mediation pathways.

**Conclusions and Relevance:**

In this cohort study of preterm infants, complete ACS courses in high-risk pregnancies were associated with a reduction in neonatal BPD, potentially mediated through multifactorial pathways. Emphasizing the importance of timely ACS completion and postnatal airway management may help optimize neonatal pulmonary outcomes.

## Key Points

**Question:**

Is there an association between antenatal corticosteroids (ACS) and bronchopulmonary dysplasia (BPD) in very preterm infants?

**Findings:**

In this cohort study of 1097 infants with gestational age less than 30 weeks, a complete course of ACS was significantly associated with a reduced risk of BPD, particularly in singleton infants delivered vaginally at 28 weeks’ to 28 weeks 6 days’ gestation.

**Meaning:**

These findings suggest that ACS could optimize pulmonary outcomes and reduce incidence of BPD in very preterm infants.

## Teaser

This cohort study investigates the association between antenatal corticosteroids and bronchopulmonary dysplasia in very preterm infants and whether respiratory distress syndrome and invasive mechanical ventilation mediate the outcomes.

## Introduction

Bronchopulmonary dysplasia (BPD) is associated with elevated risks of morbidity, mortality, and long-term multisystem comorbidities in very preterm infants.^1^ According to the 2021 China Neonatal Network database,^2^ BPD affects 29.2% of very preterm infants, with the incidence rising to 79% in those born at less than 28 weeks’ gestational age (GA).^3^ The pathogenesis of BPD involves multifactorial interactions between immature lung development and antenatal or postnatal insults, including prenatal inflammation, mechanical ventilation–induced injury, and oxidative stress. Although risk-modifying interventions exist (eg, postnatal corticosteroids to attenuate disease progression), no therapies currently reverse established BPD pathology, and the absence of curative options underscores the critical need for preventive strategies and early multidisciplinary interventions. Antenatal corticosteroids (ACS), a cornerstone of preterm care, are associated with reduced neonatal mortality and respiratory distress syndrome (RDS) risks and improved neurodevelopmental, cardiovascular, and gastrointestinal outcomes.^4,5,6,7^ Because this evidence-based antenatal intervention is associated with significantly improved perinatal outcomes in preterm infants, current clinical guidelines uniformly recommend ACS administration for pregnancies at high risk of preterm delivery between 24 weeks 0 days and 33 weeks 6 days of gestation.^8,9^

The association of ACS with enhanced fetal lung maturation and reduced incidence of neonatal RDS is well established.^10^ However, its association with BPD remains controversial,^11^ with conflicting evidence across studies.^12,13,14^ A retrospective study by Yan et al^15^ reported decreased BPD incidence in preterm cohorts with higher ACS exposure; however, this protective outcome was absent in extremely preterm infants born at less than 28 weeks’ gestation, indicating a GA-dependent heterogeneity in the ACS-BPD association. Furthermore, BPD pathogenesis is closely associated with neonatal respiratory morbidities such as RDS,^16^ with disease progression influenced by invasive mechanical ventilation (IMV) tailored to RDS severity.^17,18^ We propose that RDS and IMV lie on the causal pathway between ACS and BPD. However, prior studies have methodologically misclassified these mediators as confounders in multivariable analyses,^16,19,20,21^ potentially introducing collider bias and attenuating the observed ACS-BPD association.^22^ Notably, the causal mediating role of RDS and IMV in this association remains unexamined. Consequently, we conducted a multicenter study to systematically evaluate the association between ACS and BPD while rigorously investigating the potential mediating effects of RDS and IMV.

## Methods

### Study Design and Participants

This prospective multicenter cohort study was conducted across 28 level III neonatal intensive care units in 7 regions of China between September 1, 2019, and December 31, 2020 (eMethods in Supplement 1). The current analysis focused on BPD. This study enrolled all extremely preterm infants with a gestational age of less than 30 weeks, including both singletons and higher-order multiples. Inclusion criteria were GA less than 30 weeks, hospitalization duration more than 2 weeks, and admission to the neonatal intensive care unit (NICU) within 24 hours of birth. Exclusion criteria were congenital structural anomalies, genetic or metabolic disorders, in-hospital mortality, treatment discontinuation, or discharge against medical advice. This study adhered to the Strengthening the Reporting of Observational Studies in Epidemiology (STROBE) reporting guideline. The study was conducted in accordance with the Declaration of Helsinki^23^ and was approved by the ethics committee of the Women and Children’s Hospital affiliated to Xiamen University. Because deidentified patient data were used, a waiver of consent was obtained at all participating centers.

### Variable and Definition

We prospectively collected maternal perinatal data and neonatal characteristics. BPD was diagnosed and classified using the 2001 National Institute of Child Health and Human Development criteria,^24^ defined as oxygen dependency for at least 28 days. Severity of BPD was assessed at 36 weeks’ corrected GA or discharge as mild (no supplemental oxygen requirement), moderate (fraction of inspired oxygen [FiO_2_] <0.30), or severe (FiO_2_ ≥0.30 or need for positive pressure ventilation or mechanical ventilation). Small for gestational age (SGA) was defined as birth weight below the 10th percentile for sex and GA using the 2013 Fenton growth curves.^25^ Severe RDS (grade 3-4) was diagnosed according to criteria in *Practical Neonatology*, 5th edition,^26^ involving clinical, radiographic, and oxygenation assessments. ACS exposure was categorized as complete ACS course (2 doses of betamethasone administered intramuscularly ≥24 hours before delivery or 4 doses of dexamethasone with the last dose ≥24 hours before delivery), incomplete ACS course (1-2 doses of betamethasone or 1-4 doses of dexamethasone, with the final dose administered <24 hours before delivery), or no ACS (no documented corticosteroid administration).

### Statistical Analysis

Data analyses were performed using R, version 4.3.2 (R Project for Statistical Computing), from April 1 to May 1, 2025. Missing data were imputed via multiple chained equations using the random forest algorithm, with pooled results for baseline characteristics generated using Rubin rules (eFigure 3 in Supplement 1). Continuous variables were compared between groups using the Welch *t* test, and categorical variables were analyzed with person χ^2^ tests. Two-sided *P* < .05 defined statistical significance.

**Figure 1.**
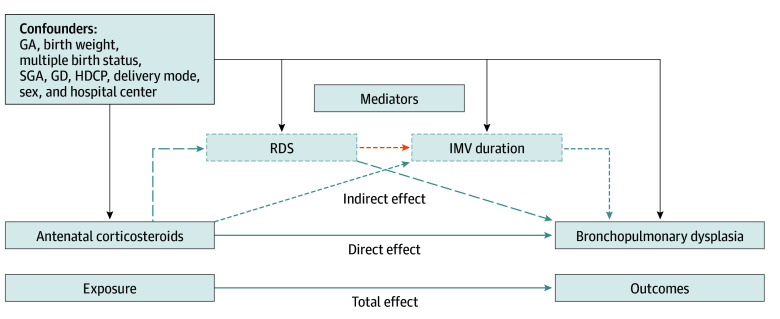
Mediation Analysis of Association Between Antenatal Corticosteroids and Bronchopulmonary Dysplasia The directed acyclic graph illustrates the mediation pathway for the association of antenatal corticosteroids (exposure) with bronchopulmonary dysplasia (outcome), mediated through respiratory distress syndrome (RDS) and duration of invasive mechanical ventilation (IMV). Total effect size represents the overall association, partitioned into direct effect (exposure to the outcome independent of mediators) and indirect effect (exposure to the outcome mediated through RDS and IMV duration). GA indicates gestational age; GD, gestational diabetes; HDCP, hypertensive disorder complicating pregnancy; SGA, small for gestational age.

Covariates were selected based on directed acyclic graphs^27^ and univariate analyses. Adjusted variables included GA, birth weight, IMV duration, sex, delivery mode (vaginal or cesarean), gestational diabetes (GD), hypertensive disorder complicating pregnancy (HDCP), SGA, plurality pregnancy, and hospital center. The exposure variable was ACS, categorized as complete course, incomplete course, or no ACS (reference category). Given that residual unmeasured confounding risks might still interfere with exposure-outcome associations despite these adjustments, we used the E-value package to evaluate unmeasured confounding effects.^28^ The primary outcome was moderate-to-severe BPD diagnosed at 36 weeks’ corrected GA. Secondary outcomes were severe RDS and IMV duration (Figure 1 and eFigure 5 in Supplement 1).

For binary outcome variables (BPD and RDS), modified Poisson regression models with robust error estimators were used to estimate adjusted risk ratios (ARRs) and 95% CIs. This approach directly quantifies risk ratios, offering advantages over logistic regression in cohort studies with high outcome incidence.^29^ For IMV duration, generalized linear models were used, with findings reported as β coefficients with 95% CIs. Three multivariable regression models were constructed with incremental covariate adjustment. Model 1 adjusted for hospital center; model 2 adjusted for GA, birth weight, and hospital center; and model 3 adjusted for GA, birth weight, plurality pregnancy, SGA, GD, HDCP, delivery mode, sex, and hospital center. All subgroup analyses for the primary outcome were predefined based on biological plausibility. Stratified analyses assessed effect heterogeneity across subgroups defined by GA, delivery mode, plurality, and sex with interaction tests performed. The Hochberg step-up procedure was applied to control the false discovery rate at α = .05 for 9 subgroup tests (singleton pregnancy, GA of 28 weeks to 28 weeks 6 days, vaginal delivery, female sex, GA of 29 weeks to 29 weeks 6 days, male sex, plurality pregnancy, cesarean delivery, and GA less than 28 weeks). We acknowledge that the exclusion of deaths may introduce survivorship bias due to shared pathologic mechanisms between extremely preterm infant death and BPD. Based on Chinese Neonatal Network data^2^ (a mortality rate of 7% [291 of 3956] for GA of 24 weeks to 29 weeks 6 days), we estimated that approximately 77 unobserved deaths might exist in the study cohort. These hypothetical data were then incorporated (total sample size needed to detect BPD was N = 1183). Subsequently, we performed an extreme-scenario analysis under a worst-case assumption (all deceased infants had BPD) and best-case assumption (all deceased infants did not have BPD) to assess survival bias. In addition, our primary analysis adjusted for hospital center as a fixed effect with robust SEs, and we used a bayesian random-intercept meta-analysis model (using weakly informative priors) to explore center-specific outcomes.

This study used a structural equation modeling–based mediation analysis framework to quantify the mechanism of ACS’s association with BPD through path analysis. The β coefficients derived from the mediation analysis represent the absolute change in the probability of the outcome. The model included (1) dual mediation paths (ACS → RDS → BPD and ACS → IMV → BPD), (2) a chained mediation path (ACS → RDS → IMV → BPD), and (3) a direct path (ACS → BPD). This study adopted the causal mediation analysis framework to decompose associations^30^: (1) direct effect, reflecting ACS’s association with BPD through nonmediation pathways; (2) indirect effect 1 (RDS pathway), reflecting the isolated mediation contribution of ACS → RDS → BPD; (3) indirect effect 2 (IMV pathway), reflecting the isolated mediation contribution of ACS → IMV → BPD; (4) indirect effect 3 (chained pathway), reflecting the cascading outcome of ACS → RDS → IMV → BPD; (5) total indirect effect, representing the combined outcome of all mediation pathways; and (6) total effect, reflecting the global association of ACS with BPD. This analytic approach delineates both independent and interdependent mediator contributions to the ACS-BPD association. To address nonnormality and enhance robustness, bias-corrected bootstrap 95% CIs were estimated using 1000 resamples. This operationalization aligns with contemporary causal mediation frameworks.^31^

## Results

### Baseline Characteristics

Among 2800 potentially eligible very preterm infants, 1703 were excluded due to incomplete data or other reasons, yielding a final analytic cohort of 1097 infants (eFigure 1 in Supplement 1). The median GA was 28.71 weeks (IQR, 27.71-29.29 weeks), median birth weight was 1150 g (IQR, 1000-1310 g), and median IMV duration was 2.0 days (IQR, 0.0-7.0 days). Overall, of 1075 infants with known sex, 476 (44%) were female and 599 (56%) were male; 346 of 1079 with known plurality status (32%) were twins or higher-order multiples, and 545 of 1077 with known delivery mode (51%) were delivered via cesarean. Of 1069 with known ACS data, 832 (78%) received ACS and 518 (48%) received a complete ACS course. Moderate-to-severe BPD occurred in 309 of 1097 infants (28%) and severe RDS in 237 of 1085 (22%). Compared with infants with no or mild BPD, those with moderate-to-severe BPD had significantly lower GA (median, 28.14 weeks [IQR, 26.86-29.14 weeks] vs 28.86 weeks [IQR, 28.11-29.32 weeks]; *P* < .001) and birth weight (median, 1040 g [IQR, 890-1200 g] vs 1200 g [IQR, 1050-1345 g]; *P* < .001). They also had higher rates of SGA (14 of 286 [5%] vs 17 of 751 [2%]; *P* = .03), plurality pregnancy (116 of 305 [38%] vs 230 of 774 [30%]; *P* = .03), severe RDS (114 of 305 [37%] vs 123 of 780 [16%]; *P* < .001), and longer IMV duration (median, 6.0 days [IQR, 2.0-19.0 days] vs 1.0 days [IQR, 0.0-5.0 days]; *P* < .001). No significant intergroup differences were observed in delivery mode, sex, GD, and HDCP (Table 1). There were significant differences in the distribution of characteristics such as sex, delivery mode, BPD, RDS, and IMV duration among preterm infants across groups with different ACS exposure levels (eTable 3 in Supplement 1).

**Table 1.** Baseline Characteristics of Very Preterm Infants

Characteristic	Infants^a^			*P* value
	Overall (n = 1097)	No or mild BPD (n = 788)	Moderate or severe BPD (n = 309)	
Gestational age, median (IQR), wk	28.71 (27.71-29.29)	28.86 (28.11-29.32)	28.14 (26.86-29.14)	<.001
Birth weight, median (IQR), g	1150 (1000-1310)	1200 (1050-1345)	1040 (890-1200)	<.001
Unknown, No.	9	7	2	NA
ACS course				
None	237/1069 (22)	156/767 (20)	81/302 (27)	.07
Incomplete	314/1069 (29)	228/767 (30)	86/302 (28)	
Complete	518/1069 (48)	383/767 (50)	135/302 (45)	
Unknown, No.	28	21	7	NA
SGA	31/1037 (3)	17/751 (2)	14/286 (5)	.03
Unknown, No.	60	37	23	NA
GD	210/1040 (20)	147/747 (20)	63/293 (22)	.51
Unknown, No.	57	41	16	NA
HDCP	169/1023 (17)	114/739 (15)	55/284 (19)	.13
Unknown, No.	74	49	25	NA
Plurality pregnancy				
Singleton	733/1079 (68)	544/774 (70)	189/305 (62)	.03
Twin	317/1079 (29)	211/774 (27)	106/305 (35)	
Triplet or higher order	29/1079 (3)	19/774 (2)	10/305 (3)	
Unknown, No.	18	14	4	NA
Delivery mode				
Vaginal	532/1077 (49)	372/771 (48)	160/306 (52)	.23
Cesarean	545/1077 (51)	399/771 (52)	146/306 (48)	
Unknown, No.	20	17	3	NA
Sex				
Female	476/1075 (44)	354/770 (46)	122/305 (40)	.08
Male	599/1075 (56)	416/770 (54)	183/305 (60)	
Unknown, No.	22	18	4	NA
Apgar score at 5 min, median (IQR)	9.00 (8.00-9.00)	9.00 (8.00-9.00)	9.00 (8.00-9.00)	<.001
Severe RDS	237/1085 (22)	123/780 (16)	114/305 (37)	<.001
Unknown, No.	12	8	4	NA
IMV duration, median (IQR), d	2.0 (0.0-7.0)	1.0 (0.0-5.0)	6.0 (2.0-19.0)	<.001
Unknown, No.	60	39	21	NA

Abbreviations: ACS, antenatal corticosteroids; BPD, bronchopulmonary dysplasia; GD, gestational diabetes; HDCP, hypertensive disorder complicating pregnancy; IMV, invasive mechanical ventilation; RDS, respiratory distress syndrome; SGA, small for gestational age.

^a^ Data are presented as number out of total number (percentage) of infants unless otherwise indicated.

### Association Between ACS and Outcomes

Spearman rank-order correlation analyses revealed inverse associations between ACS and moderate-to-severe BPD (ρ, −0.07; *P* < .001), severe RDS (ρ, −0.07; *P* = .01), and IMV duration (ρ, −0.09; *P* = .003). Positive correlations were observed between BPD and RDS (ρ, 0.24; *P* < .001) and IMV duration (ρ, 0.36; *P* < .001) (eFigure 2 in Supplement 1).

Multivariable-adjusted regression models indicated that complete ACS courses were associated with a reduction in severe RDS risk (ARR, 0.67; 95% CI, 0.51-0.88; *P* = .002) and IMV duration (β estimate, −2.003; 95% CI, −3.391 to −0.614; *P* < .001), and incomplete ACS courses also showed similar negative associations with these outcomes. Complete (ARR, 0.68; 95% CI, 0.55-0.84; *P* = .02) and incomplete (ARR, 0.78; 95% CI, 0.61-0.99; *P* = .04) ACS courses showed negative associations with moderate-to-severe BPD (Table 2). We validated the findings using the complete data from the original dataset and obtained consistent results (eTables 1 and 2 in Supplement 1).

**Table 2.** Adjusted Regression Results of Associations Between ACS and Outcomes

Model	ARR (95% CI)^a^		IMV duration, β estimate (95% CI)
	BPD	RDS	
**Model 1**			
ACS course			
None	1 [Reference]	1 [Reference]	1 [Reference]
Incomplete	0.81 (0.64-1.03)	0.76 (0.59-0.96)^b^	−1.232 (−2.107 to −0.343)^c^
Complete	0.75 (0.61-0.93)^c^	0.70 (0.56-0.87)^c^	−1.487 (−2.288 to −0.691)^d^
**Model 2**			
ACS course			
None	1 [Reference]	1 [Reference]	1 [Reference]
Incomplete	0.81 (0.65-1.01)	0.75 (0.59-0.95)^b^	−1.229 (−2.031 to −0.442)^c^
Complete	0.70 (0.57-0.85)^b^	0.85 (0.76-0.95)^c^	−1.524 (−2.241 to −0.798)^d^
**Model 3**			
ACS course			
None	1 [Reference]	1 [Reference]	1 [Reference]
Incomplete	0.78 (0.61-0.99)^b^	0.78 (0.58-1.06)	−1.683 (−3.194 to −0.172)^b^
Complete	0.68 (0.55-0.84)^b^	0.67 (0.51-0.88)^c^	−2.003 (−3.391 to −0.614)^c^

Abbreviations: ACS, antenatal corticosteroids; ARR, adjusted risk ratio; BPD, bronchopulmonary dysplasia; IMV, invasive mechanical ventilation; RDS, respiratory distress syndrome.

^a^ Model 1 adjusted for hospital; model 2 adjusted for gestational age, birth weight, and hospital; model 3 adjusted for gestational age, birth weight, plurality pregnancy, delivery mode, small for gestational age, sex, gestational diabetes, hypertensive disorder complicating pregnancy, and hospital.

^b^ *P* < .05.

^c^ *P* < .01.

^d^ *P* < .001.

### Sensitivity Analyses

**Figure 2.**
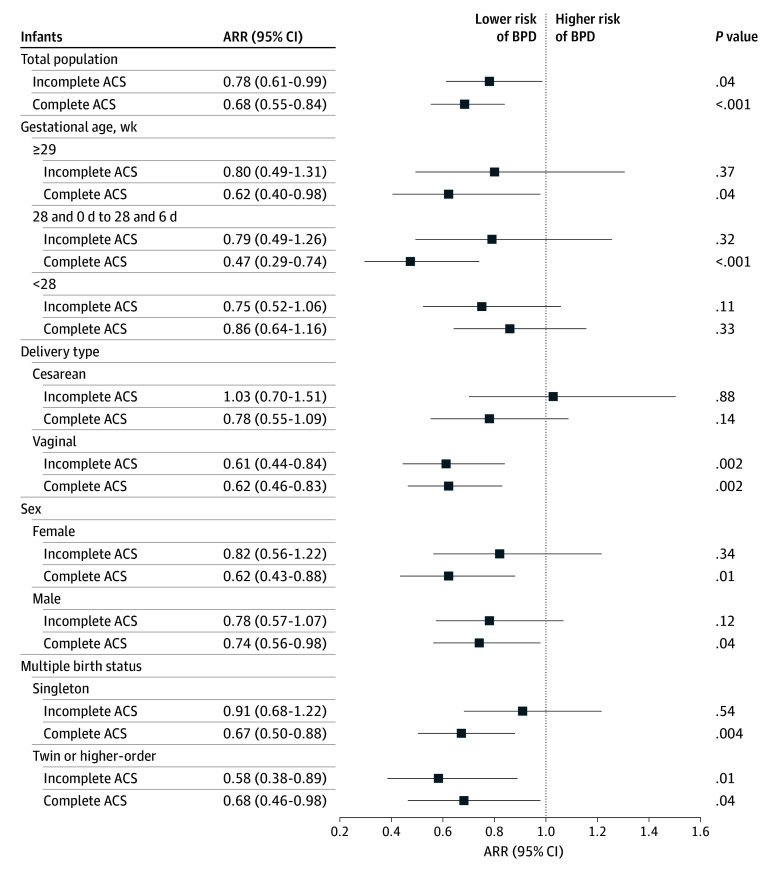
Association of Incomplete and Complete Courses of Antenatal Corticosteroids (ACS) With Bronchopulmonary Dysplasia (BPD) in Very Preterm Infants Stratified by Demographic Variables The reference category was no ACS. 95% CIs are presented without adjustment for multiple comparisons. *P* values were adjusted using the Hochberg step-up procedure to control the false discovery rate across 9 prespecified subgroup tests (singleton pregnancy, GA of 28 weeks to 28 weeks 6 days, vaginal delivery, female sex, GA of 29 weeks to 29 weeks 6 days, male sex, plurality pregnancy, cesarean delivery, and GA less than 28 weeks). ARR indicates adjusted risk ratio.

Stratified analyses demonstrated differential associations of complete ACS courses with BPD risk. After Hochberg adjustment for multiple comparisons (9 subgroups), complete ACS courses remained significantly associated with reduced BPD risk in singletons (ARR, 0.67; 95% CI, 0.50-0.88; *P* = .004; adjusted *P* = .004), infants born at 28 weeks to 28 weeks 6 days of gestation (ARR, 0.47; 95% CI, 0.29-0.74; *P* = .001; adjusted *P* = .01), and vaginal births (ARR, 0.62; 95% CI, 0.46-0.83; *P* = .002; adjusted *P* = .01). No significant associations were observed for the subgroups born by cesarean delivery or with GA of less than 28 weeks (Figure 2 and eTable 4 in Supplement 1). The sensitivity analysis indicated that the association of a complete ACS course with protection against BPD remained robust whether all deaths were classified as BPD (ARR, 0.76; 95% CI, 0.62-0.92; *P* = .01) or non-BPD (ARR, 0.79; 95% CI, 0.63-0.99; *P* = .04) (eFigure 4 in Supplement 1). The bayesian random-intercept meta-analysis suggested a negative association between a complete course of ACS and odds of BPD (odds ratio [OR], 0.65; 95% CI, 0.44-0.97). No statistically significant differences in baseline risk and comparisons of intergroup differences were observed between groups. Although statistically significant heterogeneity in baseline risk existed between centers (τ^2^, 0.721; 95% CI, 0.342-1.599; *P* > .99 for τ^2^ > 0), this heterogeneity explained only 18% of the total variation (*I*^2^, 0.18; 95% CI, 0.09-0.33) (eTable 5 in Supplement 1).

### Mediating Role of RDS and IMV Duration in the ACS-BPD Association

The model suggested a clinically meaningful total BPD reduction associated with ACS, corresponding to an absolute reduction in the risk of BPD (β, −0.050; 95% CI, −0.081 to −0.017; *P* = .002). This association was partitioned into a direct association with risk reduction (β, −0.031; 95% CI, −0.061 to −0.001; *P* = .04) and a total indirect association with risk reduction (β = −0.019; 95% CI, −0.032 to −0.007; *P* = .002), which accounted for 40% of the observed association and suggested the possible presence of predominant mediation mechanisms. Of note, the indirect association operated through several significant pathways: ACS was associated with a reduced risk of RDS, which subsequently mediated a reduction in BPD incidence (β, −0.007; 95% CI, −0.014 to −0.002; *P* = .02). Furthermore, ACS was associated with decreased duration of IMV, and this reduction mediated an attenuation of BPD risk (β, −0.009; 95% CI, −0.019 to −0.001; *P* = .04). In addition, serial mediation analyses revealed a significant pathway wherein ACS mitigated BPD risk through sequential mechanisms: reduced RDS was associated with lower IMV duration, ultimately diminishing BPD risk (β, −0.003; 95% CI, −0.006 to −0.001; *P* = .01) (Table 3). This suggested that an estimated 62% of ACS’s protective effect might be direct and 38% might operate through indirect pathways: independent RDS mediation (14%), independent IMV mediation (18%), and a serial mediation (ACS → RDS → IMV → BPD) (6%).

**Table 3.** Direct and Indirect Associations of ACS With BPD via RDS and IMV Duration

Pathway^a^	β Estimate (SE) [95% CI]^b^	*Z* value	*P* value
Direct effect			
ACS → BPD	−0.031 (0.015) [−0.061 to −0.001]	−2.05	.04
Indirect effect			
ACS → RDS → BPD	−0.007 (0.003) [−0.014 to −0.002]	−2.31	.02
ACS → IMV → BPD	−0.009 (0.005) [−0.019 to −0.001]	−2.04	.04
ACS → RDS → IMV → BPD	−0.003 (0.001) [−0.006 to −0.001]	−2.50	.01
Total indirect effect	−0.019 (0.006) [−0.032 to −0.007]	−3.11	.002
Total effect	−0.050 (0.016) [−0.081 to −0.017]	−3.07	.002

Abbreviations: ACS, antenatal corticosteroids; BPD, bronchopulmonary dysplasia; IMV, invasive mechanical ventilation; RDS, respiratory distress syndrome.

^a^ The total indirect effect size represents the sum of all mediated pathways. Model fit indices: χ^2^_1_ = 1.203 (*P* = .27); standardized root mean square residual = 0.003; root mean square error of approximation = 0.014; comparative fit index = 1.000; Tucker Lewis index = 0.994.

^b^ Bootstrap 95% CIs based on 1000 resamples (percentile method).

## Discussion

This multicenter cohort study provides novel evidence supporting the association between ACS and reduced BPD risk in very preterm infants. Of importance, we report the first quantification, to our knowledge, of the serial mediation pathway ACS → RDS → IMV → BPD, which appeared to account for 6% of the total protective effect of ACS.

BPD manifests through distinct histopathologic phenotypes, including pulmonary fibrosis, atelectasis, and cystic changes. In infants born before 29 weeks’ gestation, this condition primarily features impaired parenchymal development and dysregulated vascular growth.^32^ Central to BPD pathogenesis is inflammation-driven disruption of lung development. Prenatal exposure to intrauterine inflammation is a key driver of this process, which disrupts alveolarization and angiogenesis, triggers neutrophil and macrophage infiltration in the developing lung, and is mechanistically linked to BPD onset.^33^ These pathophysiologic mechanisms underscore the imperative for antenatal prevention and optimized postnatal respiratory management.

RDS and IMV constitute established risk factors for BPD,^34,35^ both primarily stemming from pulmonary immaturity in prematurity. Preterm infants with RDS frequently require mechanical ventilation and oxygen therapy—interventions that expose immature airways to hyperoxia and barotrauma. This is associated with inflammatory cytokine release, oxidative stress, arrested lung development, and airway remodeling, culminating in BPD.^36^ ACS promotes fetal lung maturation through multiple mechanisms, including suppression of pulmonary cell proliferation, shortening of the gas diffusion distance, enhanced pulmonary surfactant synthesis, and accelerated airway fluid clearance at birth.^5,37^ Additionally, Crawshaw et al^38^ demonstrated that combined administration of surfactant and betamethasone synergistically improved functional residual capacity recruitment in preterm models, implying that ACS not only mitigates RDS risk but also enhances the therapeutic efficacy of pulmonary surfactant.^39^ These actions could explain ACS’s apparent protective effects against RDS while reducing IMV requirements,^5,8,40,41,42,43^ thereby attenuating barotrauma, oxidative stress, and downstream pathologic cascades. Our mediation analysis suggested that an estimated 38% of ACS’s protective effect might operate through indirect pathways: independent RDS mediation (14%), independent IMV mediation (18%), and a serial mediation ACS → RDS → IMV → BPD (6%).

Glucocorticoid-mediated anti-inflammatory effects persist into the neonatal period, potentially providing sustained tissue protection.^44^ In a prenatal endotoxin-induced BPD model, ACS was associated with significantly improved pulmonary mechanics (15.3% reduction in total lung resistance [*P* < .05], 9.5% increased compliance [*P* < .05]) while preserving alveolarization and vascular growth—correlating with superior respiratory outcomes.^45^ Through inflammation suppression, ACS may help preserve normal developmental processes,^46,47^ which could help explain its dominant direct effect (accounting for an estimated 62% of total effects). Of note, the magnitude of the direct association suggests the possibility of alternative pathways—such as suppression of intrauterine inflammation and promotion of alveolar-vascular development—that might operate independently of RDS and IMV. Altogether, ACS likely mediates lung protection in prematurity through dual mechanisms^48^: both anti-inflammatory actions and promaturation effects.

A dose-response relationship has been proposed for ACS efficacy. Neonates born to mothers receiving no ACS exhibited 3-fold increased odds of BPD compared with those exposed to complete courses, while incomplete ACS courses did not significantly reduce the risk of BPD.^35^ Consistent with these findings, our study demonstrated that ACS courses were significantly associated with reduced moderate-to-severe BPD risk and complete ACS courses were associated with superior risk reduction with tighter 95% CIs. The 2022 European guidelines for RDS management emphasize accurately predicting preterm delivery timing in high-risk pregnancies.^49^ Women at imminent risk of extreme preterm birth should receive interventions to prolong gestation, ensuring completion of ACS courses for maximal pulmonary benefit.^49^

This study indicated possible enhanced efficacy of ACS in vaginally delivered singletons at 28 weeks to 28 weeks 6 days of gestation. While significant response heterogeneity existed across subgroups, similar to the findings of previous studies,^10,50,51,52,53^ this does not negate ACS’s potential benefits in other preterm infants. The attenuated protection observed in certain populations could reflect the survival paradox (improved post-ACS viability increases the absolute number of BPD-susceptible infants) and unmeasured postnatal confounders (eg, ventilator-induced injury, sepsis, or prolonged oxygen therapy) that may persistently impair lung development,^32^ potentially masking ACS-related protection.

### Strengths and Limitations

Strengths of our study include the recruitment of nearly all eligible preterm infants from 28 level III NICUs; the acquisition of a large multicenter sample minimized selection bias and enhanced population representativeness. Second, through rigorous adjustment for confounding variables, we validated the associations between ACS and risks of BPD, RDS, and IMV. Stratified analyses further elucidated heterogeneity in ACS protective outcomes across clinically relevant subgroups. In addition, this study both supports the primary role of indirect pathways and proposes novel sequential (ACS → RDS → IMV → BPD) mechanisms that might underlie ACS-associated BPD risk reduction.

Several limitations warrant consideration. Despite multivariable adjustments, residual confounding from unmeasured factors may persist (eTable 4 in Supplement 1). While mediation analyses provided mechanistic insights, the observational design precludes definitive causal inferences, necessitating experimental validation. Additionally, the limited sample size of extremely preterm infants may have obscured ACS effects in this high-risk subgroup, potentially underestimating the clinical utility of ACS.

## Conclusions

This cohort study of preterm infants found that complete ACS courses in high-risk pregnancies may be associated with a reduction in neonatal BPD, potentially mediated through multifactorial pathways. Emphasizing the importance of timely ACS completion and postnatal airway management may help optimize neonatal pulmonary outcomes.

## Notes

Supplement 1. **eTable 1.** Baseline Characteristics of Original Data **eTable 2.** Effect of ACS on BPD in Very Preterm Infants With Complete Data **eTable 3.** Baseline Characteristics of the Study Population According to ACS Exposure Levels **eTable 4.** Results of Multiple Test Correction for Hochberg Method in Subgroup Analysis **eTable 5.** The Center-Specific Effects Analysis by Bayesian Random-Intercept Meta-Analysis Model **eFigure 1.** Flowchart **eFigure 2.** Correlation Matrix of Variables **eFigure 3.** Assessment of Missing Values **eFigure 4.** Extreme Scenario Analysis **eFigure 5.** Direct Acyclic Graph Designed Using Dagitty **eMethods.** Demographic Distribution of the Original Study Cohort

Supplement 2.
Chinese Multicenter EUGR Collaborative Group members

Supplement 3.
Data Sharing Statement
