# Supplementary material for: Antenatal Corticosteroids and Bronchopulmonary Dysplasia in Very Preterm Infants
Source: JAMA Netw Open. 2025 Nov 26;8(11):e2545606. doi: 10.1001/jamanetworkopen.2025.45606 (PMC12658673; doi:10.1001/jamanetworkopen.2025.45606)
Supplement: Supplement 1. — eTable 1. Baseline Characteristics of Original Data eTable 2. Effect of ACS on BPD in Very Preterm Infants With Complete Data eTable 3. Baseline Characteristics of the Study Population According to ACS Exposure Levels eTable 4. Results of Multiple Test Correction for Hochberg Method in Subgroup Analysis eTable 5. The Center-Specific Effects Analysis by Bayesian Random-Intercept Meta-Analysis Model eFigure 1. Flowchart eFigure 2. Correlation Matrix of Variables eFigure 3. Assessment of Missing Values eFigure 4. Extreme Scenario Analysis eFigure 5. Direct Acyclic Graph Designed Using Dagitty eMethods. Demographic Distribution of the Original Study Cohort [file jamanetwopen-e2545606-s001.pdf]

Supplementary Online Content

Gao L, Zheng Z, Lin X-Z, Shen W. Antenatal corticosteroids and bronchopulmonary dysplasia in very preterm infants. *JAMA Netw Open*. 2025;8(11):e2545606. doi:10.1001/jamanetworkopen.2025.45606

eTable 1. Baseline Characteristics of Original Data
eTable 2. Effect of ACS on BPD in Very Preterm Infants With Complete Data
eTable 3. Baseline Characteristics of the Study Population According to ACS Exposure Levels
eTable 4. Results of Multiple Test Correction for Hochberg Method in Subgroup Analysis
eTable 5. Center-Specific Effects Analysis by Bayesian Random-Intercept Meta-Analysis Model
eFigure 1. Flowchart
eFigure 2. Correlation Matrix of Variables
eFigure 3. Assessment of Missing Values
eFigure 4. Extreme Scenario Analysis
eFigure 5. Direct Acyclic Graph Designed Using Dagitty
eMethods. Demographic Distribution of the Original Study Cohort

This supplementary material has been provided by the authors to give readers additional information about their work.

© 2025 Gao L et al. *JAMA Network Open*.

eTable 1. Baseline characteristics of original data

Characteristic	Overall, N = 1097	No or Mild BPD, n = 788	Moderate or Severe BPD, n = 309	p-value
Gestational age, Median (IQR), w	28.71 (27.71 – 29.29)	28.86 (28.11 – 29.32)	28.14 (26.86 – 29.14)	<0.001
Birth weight, Median (IQR), g	1,150.00 (1,000.00 – 1,310.00)	1,200.00 (1,050.00 – 1,345.00)	1,040.00 (890.00 – 1,200.00)	<0.001
Unknown	9	7	2	
ACS, n (%)				0.07
No	237 (22)	156 (20)	81 (27)	
Incomplete	314 (29)	228 (30)	86 (28)	
Complete	518 (48)	383 (50)	135 (45)	
Unknown	66	61	5	
SGA, n (%)	31 (3.0)	17 (2.3)	14 (4.9)	0.03
Unknown	60	37	23	
GDM, n (%)	210 (20)	147 (20)	63 (22)	0.51
Unknown	57	41	16	
HDCP, n (%)	169 (17)	114 (15)	55 (19)	0.13
Unknown	74	49	25	
Multiple, n (%)				0.03
Singleton	733 (68)	544 (70)	189 (62)	
Twin	317 (29)	211 (27)	106 (35)	
Triplet and higher-order	29 (2.7)	19 (2.5)	10 (3.3)	
Unknown	18	14	4	
Delivery mode, n (%)				0.23
Vaginal	532 (49)	372 (48)	160 (52)	
Caesarean section	545 (51)	399 (52)	146 (48)	
Unknown	20	17	3	

© 2025 Gao L et al. *JAMA Network Open*.

Characteristic	Overall, N = 1097	No or Mild BPD, n = 788	Moderate or Severe BPD, n = 309	p-value
Sex, n (%)				0.08
Female	476 (44)	354 (46)	122 (40)	
Male	599 (56)	416 (54)	183 (60)	
Unknown	22	18	4	
5 minute Apgar, Median (IQR)	9.00 (8.00 – 9.00)	9.00 (8.00 – 9.00)	9.00 (8.00 – 9.00)	<0.001
Severe RDS, n (%)	237 (22)	123 (16)	114 (37)	<0.001
Unknown	12	8	4	
IMV duration, Median (IQR), d	2.00 (0.00 – 7.00)	1.00 (0.00 – 5.00)	6.00 (2.00 – 19.00)	<0.001
Unknown	60	39	21	

Notes: ACS: antenatal corticosteroids; BPD: bronchopulmonary dysplasia; RDS: respiratory distress syndrome; SGA: small for gestation; GDM: gestational diabetes mellitus; HDCP: hypertensive disorder complicating pregnancy; IMV: invasive mechanical ventilation

© 2025 Gao L et al. *JAMA Network Open*.

eTable 2. Effect of ACS on BPD in very preterm infants with complete data

		BPD		RDS		IMV duration	
		**aRR**	**95% CI**	**aRR**	**95% CI**	**Estimate**	**95% CI**
ACS	No	Ref		Ref		Ref	
	Incomplete	0.77	0.57-1.04	0.68*	0.48-0.96	-1.98*	-3.69 ~ -0.26
	Complete	0.73*	0.56-0.95	0.62**	0.45-0.85	-1.90*	-3.48 ~ -0.31

Notes: ACS: antenatal corticosteroids; BPD: bronchopulmonary dysplasia; RDS: respiratory distress syndrome; IMV: invasive mechanical ventilation. Adjust variables: gestation age, birth weight, delivery, small for gestation, sex, GDM, HDCP, and hospital were adjusted. ***p<0.001; **p<0.01; *p<0.05

© 2025 Gao L et al. *JAMA Network Open*.

eTable 3. Baseline characteristics of the study population according to ACS exposure levels

Characteristic	Overall, n = 1106	No, n = 243	Incomplete ACS, n = 324	Complete ACS, n = 539	p-value
Gestational age, Median (IQR), w	28.71 (27.71 – 29.29)	28.71 (27.71 – 29.29)	28.71 (27.71 – 29.29)	28.71 (27.86 – 29.29)	0.69
Birth weight, Median (IQR), g	1,150.00 (1,000.00 – 1,310.00)	1,160.00 (1,008.50 – 1,345.00)	1,170.00 (1,028.75 – 1,330.00)	1,150.00 (950.00 – 1,290.00)	0.05
Sex, n (%)					0.02
Female	487 (44)	97 (40)	130 (40)	260 (48)	
Male	619 (56)	146 (60)	194 (60)	279 (52)	
SGA, n (%)	34 (3.1)	4 (1.6)	8 (2.5)	22 (4.1)	0.14
Delivery mode, n (%)					<0.001
Vaginal	547 (49)	140 (58)	183 (56)	224 (42)	
Caesarean section	559 (51)	103 (42)	141 (44)	315 (58)	
5 minute Apgar, Median (IQR)	9.00 (8.00 – 9.00)	9.00 (8.00 – 9.00)	9.00 (8.00 – 9.00)	9.00 (8.00 – 9.00)	0.49
BPD, n (%)	311 (28)	84 (35)	89 (27)	138 (26)	0.03
HDCP, n (%)	182 (16)	27 (11)	51 (16)	104 (19)	0.02
GDM, n (%)	218 (20)	49 (20)	61 (19)	108 (20)	0.89
Multiple, n (%)					0.01
Singleton	747 (68)	183 (75)	212 (65)	352 (65)	
Twin	329 (30)	58 (24)	98 (30)	173 (32)	
Triplet and higher-order	30 (2.7)	2 (0.8)	14 (4.3)	14 (2.6)	
IMV duration, Median (IQR), d	2.00 (0.00 – 6.23)	3.00 (0.00 – 8.00)	2.00 (0.00 – 6.00)	2.00 (0.00 – 6.00)	0.004
Severe RDS, n (%)	242 (22)	67 (28)	71 (22)	104 (19)	0.04

Notes: ACS: antenatal corticosteroids; BPD: bronchopulmonary dysplasia; RDS: respiratory distress syndrome; SGA: small for gestation; GDM: gestational diabetes mellitus; HDCP: hypertensive disorder complicating pregnancy; IMV: invasive mechanical ventilation

© 2025 Gao L et al. *JAMA Network Open*.

eTable 4. Results of multiple test correction for Hochberg method in subgroup analysis

Subgroup	aRR (95% CI)	Unadjusted_P	Hochberg_Critical	Adjusted_P	Significant
Singleton	0.67(0.50-0.88)	0.0004	0.01	0.004	TRUE
GA 28-28^{+6}	0.47(0.29-0.74)	0.001	0.01	0.01	TRUE
Vaginal	0.62(0.46-0.83)	0.002	0.01	0.01	TRUE
Female	0.61(0.43-0.88)	0.01	0.01	0.06	FALSE
GA 29-29^{+6} w	0.62(0.40-0.98)	0.04	0.02	0.12	FALSE
Male	0.74(0.56-0.98)	0.04	0.01	0.12	FALSE
Multiple	0.68(0.46-0.98)	0.04	0.02	0.12	FALSE
Cesarean	0.78(0.55-1.09)	0.14	0.03	0.28	FALSE
GA<28 w	0.86(0.64-1.16)	0.33	0.05	0.33	FALSE

© 2025 Gao L et al. *JAMA Network Open*.

eTable 5. Center-specific effects analysis by Bayesian random-intercept meta-analysis model

Analytical dimensions	Estimate	Lower	Upper	P-value
**Overall ACS effects, OR(95%CI)***				
Incomplete	0.71	0.46	1.07	
Complete	0.65	0.44	0.97	
**Baseline Risk, β (95% CrI)**				
Large center (>30)	0.14	-0.28	0.56	
Medium center (15-30)	-0.14	-0.54	0.26	
Small center (<15)	-0.21	-0.79	0.37	
**Differences in group, β (95% CrI)**				
Large vs small	0.50	-0.60	1.64	
Large vs medium	0.29	-0.55	1.16	
Medium vs small	0.21	-1.08	1.53	
**Heterogeneity indicators**				
τ^2	0.72	0.34	1.60	1.00#
I^2	0.18	0.09	0.33	1.00#
Heterogeneity test				<0.001

Notes:* The parametes was obtained by exponential transformation of the estimate of the Bayesian random-intercept meta-analysis model. #The posterior probability of Bayesian test. ACS: antenatal corticosteroids;

© 2025 Gao L et al. *JAMA Network Open*.

eFigure 1. Flowchart

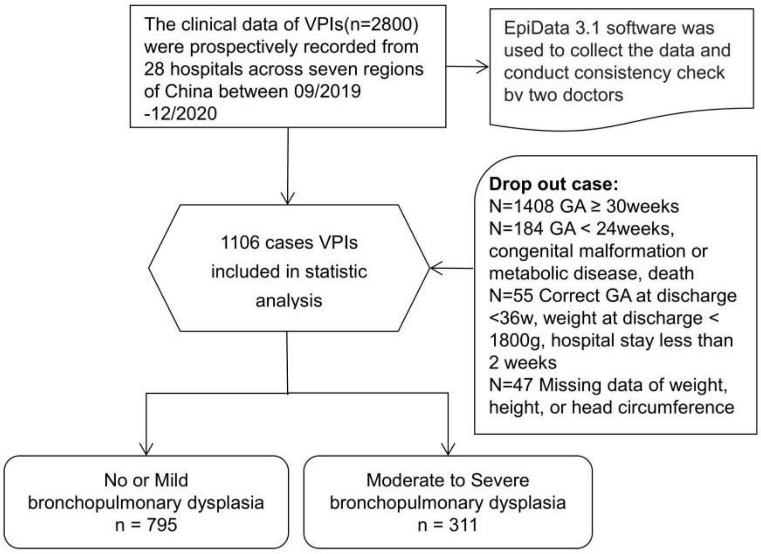

Note: GA: gestational age; VPIs: very preterm infants.

© 2025 Gao L et al. *JAMA Network Open*.

eFigure 2. Correlation matrix of variables

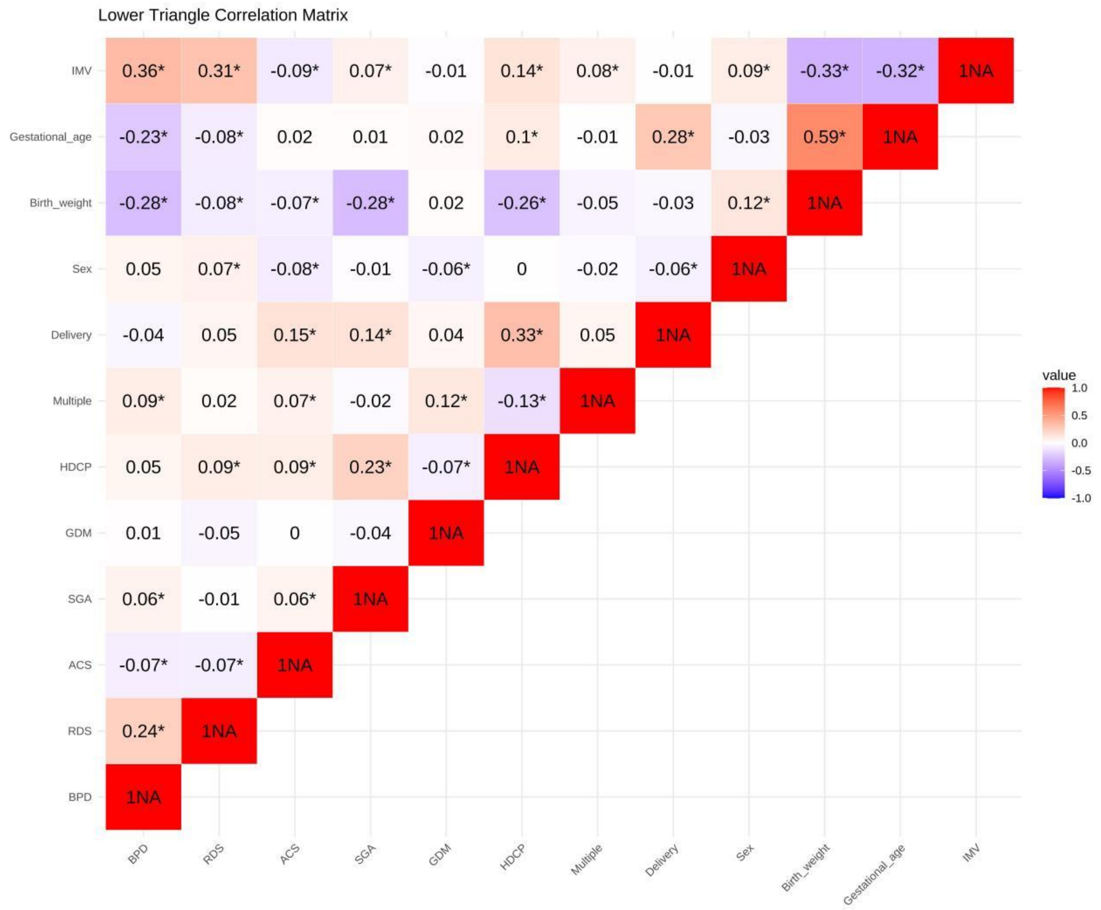

Notes: ACS: antenatal corticosteroids; BPD: bronchopulmonary dysplasia; RDS: respiratory distress syndrome; SGA: small for gestation; GDM: gestational diabetes mellitus; HDCP: hypertensive disorder complicating pregnancy; IMV: invasive mechanical ventilation.

eFigure 3. Assessment of missing values.

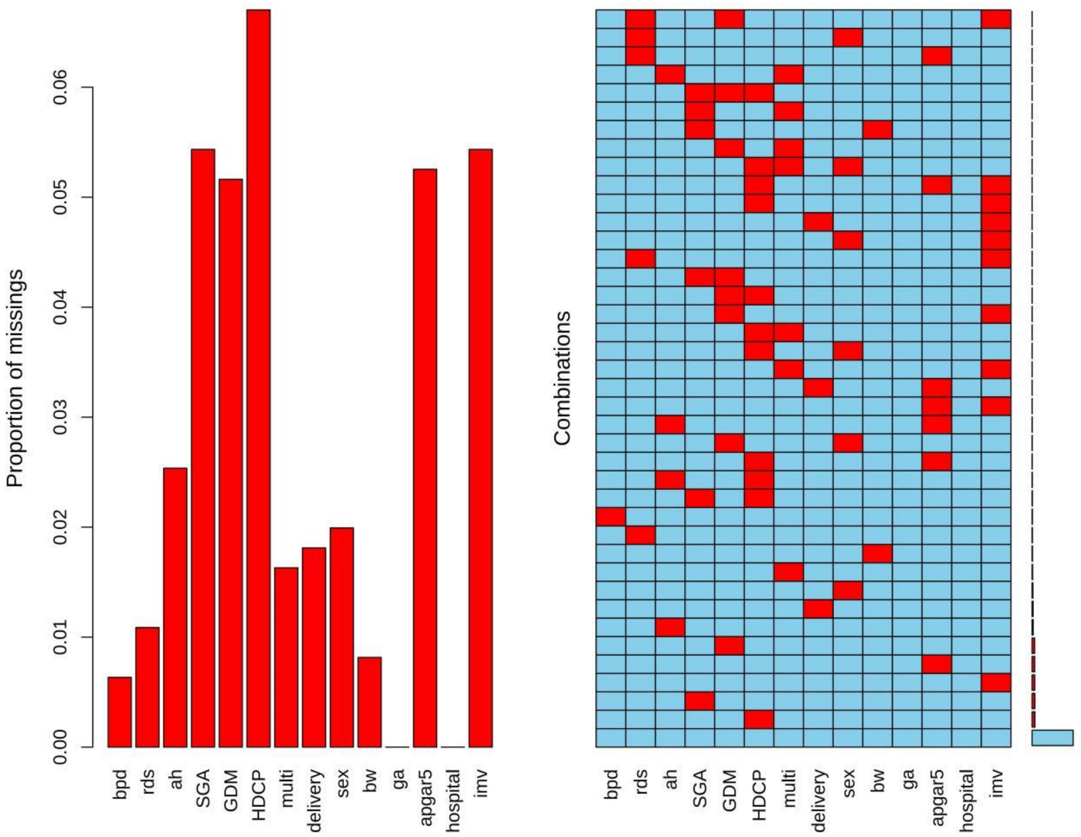

© 2025 Gao L et al. *JAMA Network Open*.

eFigure 4. Extreme scenario analysis

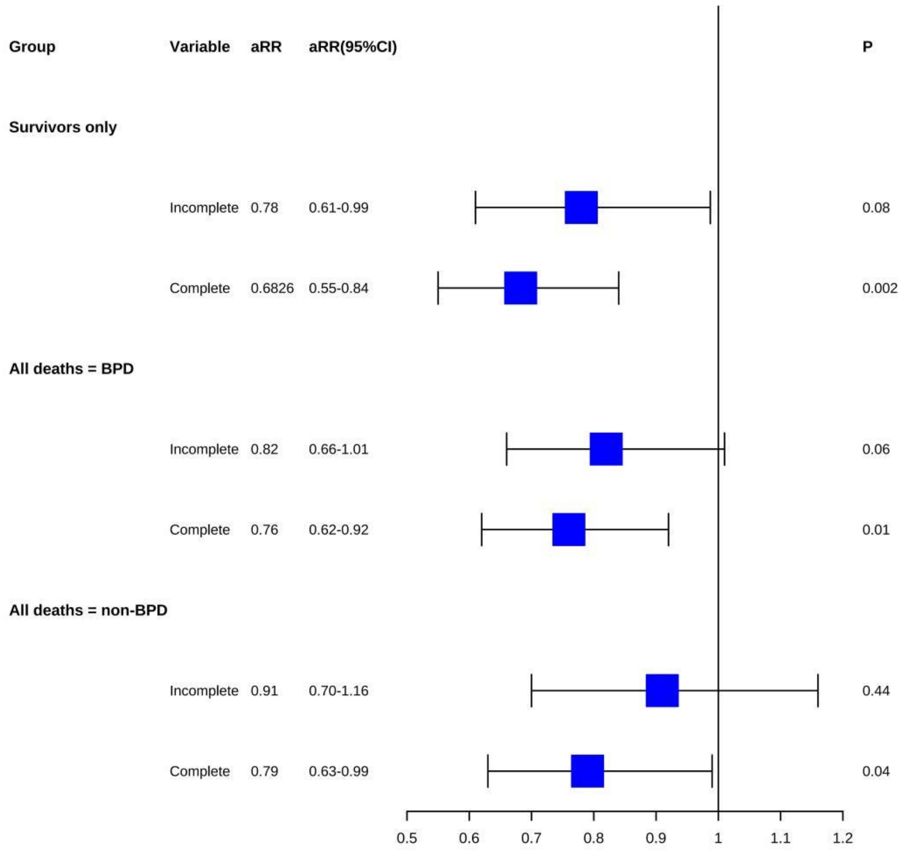

© 2025 Gao L et al. *JAMA Network Open*.

eFigure 5. Direct acyclic graph designed using Dagitty.

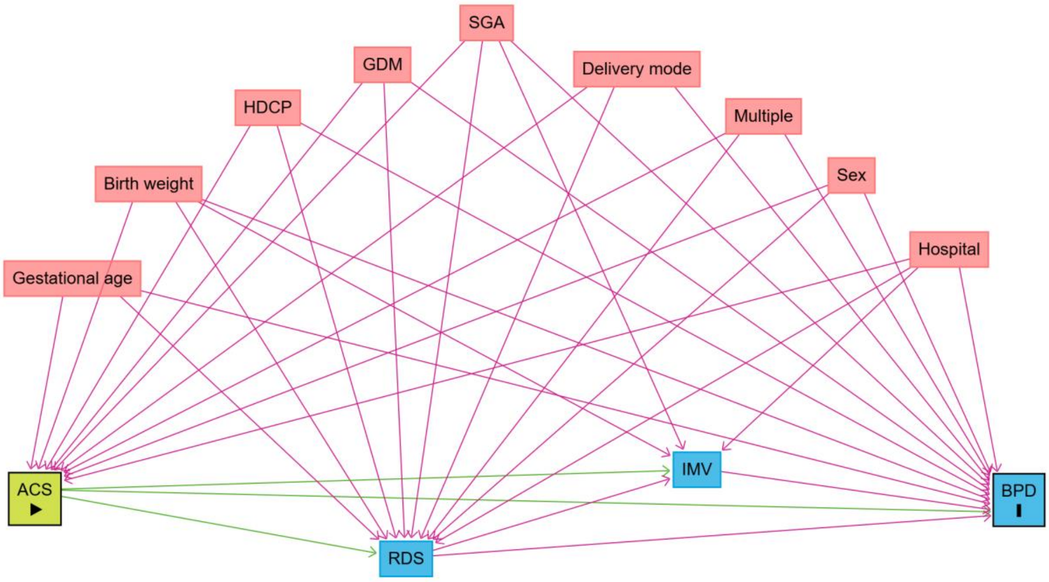

Notes: ACS: antenatal corticosteroids; BPD: bronchopulmonary dysplasia; RDS: respiratory distress syndrome; SGA: small for gestation; GDM: gestational diabetes mellitus; HDCP: hypertensive disorder complicating pregnancy; IMV: invasive mechanical ventilation

© 2025 Gao L et al. *JAMA Network Open*.

eMethods. Demographic distribution of the original study cohort

The original study is a multi-center prospective survey from September 2019 to December 2020. The data were obtained from 28 tertiary hospitals in 7 regions of Northeast, North, East, Central, South, Northwest, and Southwest China, including 13 tertiary general hospitals, 11 children's hospitals, and 4 maternal and child health hospitals.

A total of 2,514 VPIs were finally included for statistical analysis. Among them, there were 1, 378 (54.81%) male infants and 1, 136 (45.19%) female infants, with gestational age of 30.1 (28.9, 31.1) weeks and birth weight of 1, 330.5 (1,130.0, 1,540.0) g. They had gestational age of 36.9 (36.0, 37.9) weeks and weight of 2,240.0 (2,040.0, 2, 496.3) g at discharge.

Among the 2,514 VPIs, 1,694 (67.38%) were singleton births, 820 (32.62%) were twin or multiple births; 975 (38.78%) were born by vaginal delivery, and 1,539 (61.22%) were born by cesarean section. 77.9%(1958/2514) received ACS, and 48.7%(1224/2514) received a complete ACS course. Moderate-to-severe BPD occurred in 16.2% (407/2,514), and severe nRDS in 15% (384/2,514).

© 2025 Gao L et al. *JAMA Network Open*.
