# Supplementary material for: Antenatal Corticosteroids and Bronchopulmonary Dysplasia in Very Preterm Infants
Source: JAMA Netw Open. 2025 Nov 26;8(11):e2545606. doi: 10.1001/jamanetworkopen.2025.45606 (PMC12658673; doi:10.1001/jamanetworkopen.2025.45606)
Supplement: Supplement 2. — Chinese Multicenter EUGR Collaborative Group members [file jamanetwopen-e2545606-s002.pdf]

Supplemental Online Content: Nonauthor Collaborators
*First name, last name, and suffix (if applicable) are required and will appear in PubMed.

*Group Name(s): The Chinese Multicenter EUGR Collaborative Group										
***First Name and Middle Initial(s)**	***Last Name**	***Suffix (eg, Jr, III)**	Academic Degrees	Institution	Location (city, state/province, country)	Role or Contribution, eg, chair, principal investigator	Group (if more than 1 Group listed in the byline) and/or Subgroup (eg, Steering Committee)			
Xin zhu	Lin		Bachelor	Women and Children's Hospital, Scho	Xiamen, Fujian, China	Chair	The Chinese Multicenter EUGR Collaborative Group			
Wei	Shen		Doctor	Women and Children's Hospital, Scho	Xiamen, Fujian, China	Principal investigator	The Chinese Multicenter EUGR Collaborative Group			
Fan	Wu		Doctor	Third Affiliated Hospital of Guangzho	Guangzhou, Guangdong, China	Principal investigator	The Chinese Multicenter EUGR Collaborative Group			
Qian xin	Tian		Doctor	Third Affiliated Hospital of Guangzho	Guangzhou, Guangdong, China	Principal investigator	The Chinese Multicenter EUGR Collaborative Group			
Jian	Mao		Doctor	Shengjing Hospital of China Medical U	Shenyang, Liaoning, China	Principal investigator	The Chinese Multicenter EUGR Collaborative Group			
Yuan	Yuan		Doctor	Shengjing Hospital of China Medical U	Shenyang, Liaoning, China	Principal investigator	The Chinese Multicenter EUGR Collaborative Group			
Ling	Liu		Doctor	Guiyang Maternal and Child Health H	Guiyang, Guizhou, China	Principal investigator	The Chinese Multicenter EUGR Collaborative Group			
Bi zhen	Shi		Doctor	Guiyang Maternal and Child Health H	Guiyang, Guizhou, China	Principal investigator	The Chinese Multicenter EUGR Collaborative Group			
Xiao mei	Tong		Doctor	Peking University Third Hospital	Beijing, China	Principal investigator	The Chinese Multicenter EUGR Collaborative Group			
Jing hui	Zhang		Doctor	Peking University Third Hospital	Beijing, China	Principal investigator	The Chinese Multicenter EUGR Collaborative Group			
Rong	Zhang		Doctor	Pediatric Hospital of Fudan University	Shanghai, China	Principal investigator	The Chinese Multicenter EUGR Collaborative Group			
Yan	Zhu		Doctor	Pediatric Hospital of Fudan University	Shanghai, China	Principal investigator	The Chinese Multicenter EUGR Collaborative Group			
Xiu zhen	Ye		Master	Guangdong Province Maternal and C	Guangzhou, Guangdong, China	Principal investigator	The Chinese Multicenter EUGR Collaborative Group			
Jing jing	Zou		Master	Guangdong Province Maternal and C	Guangzhou, Guangdong, China	Principal investigator	The Chinese Multicenter EUGR Collaborative Group			
Yin ping	Qiu		Bachelor	General Hospital of Ningxia Medical U	Yinchuan, Ningxia, China	Principal investigator	The Chinese Multicenter EUGR Collaborative Group			
Yu huai	Li		Master	General Hospital of Ningxia Medical U	Yinchuan, Ningxia, China	Principal investigator	The Chinese Multicenter EUGR Collaborative Group			
Li	Ma		Doctor	Children's Hospital of Hebei Provinc	Shijiazhuang, Hebei, China	Principal investigator	The Chinese Multicenter EUGR Collaborative Group			
Shua hua	Liu		Master	Children's Hospital of Hebei Provinc	Shijiazhuang, Hebei, China	Principal investigator	The Chinese Multicenter EUGR Collaborative Group			
Rui	Cheng		Doctor	Children's Hospital of Nanjing Medica	Nanjing, Jiangsu, China	Principal investigator	The Chinese Multicenter EUGR Collaborative Group			
Ying	Xu		Master	Children's Hospital of Nanjing Medica	Nanjing, Jiangsu, China	Principal investigator	The Chinese Multicenter EUGR Collaborative Group			
Hui	Wu		Doctor	The First Hospital of Jilin University	Changchun, Jilin, China	Principal investigator	The Chinese Multicenter EUGR Collaborative Group			
Wen li	Zhou		Master	The First Hospital of Jilin University	Changchun, Jilin, China	Principal investigator	The Chinese Multicenter EUGR Collaborative Group			
Dong mei	Chen		Master	Quanzhou Maternity and Children's H	Quanzhou, Fujian, China	Principal investigator	The Chinese Multicenter EUGR Collaborative Group			
Zhi yong	Liu		Master	Quanzhou Maternity and Children's H	Quanzhou, Fujian, China	Principal investigator	The Chinese Multicenter EUGR Collaborative Group			
Ling	Chen		Doctor	Tongji Hospital, Tongji Medical Colleg	Wuhan, Hubei, China	Principal investigator	The Chinese Multicenter EUGR Collaborative Group			
Ping	Xu		Master	Liaocheng people's hospital	Liaocheng, Shandong, China	Principal investigator	The Chinese Multicenter EUGR Collaborative Group			
Hua	Mei		Master	The Affiliate Hospital of Inner Mongo	Hohhot, Inner Mongolia, China	Principal investigator	The Chinese Multicenter EUGR Collaborative Group			
San nan	Wang		Master	Suzhou Municipal Hospital	Suzhou, Jiangsu, China	Principal investigator	The Chinese Multicenter EUGR Collaborative Group			
Fa lin	Xu		Doctor	The Third Affiliated Hospital of Zheng	Zhengzhou, Henan, China	Principal investigator	The Chinese Multicenter EUGR Collaborative Group			
Rong	Ju		Doctor	Chengdu Women' and Children's Cen	Chengdu, Sichuan, China	Principal investigator	The Chinese Multicenter EUGR Collaborative Group			
Xiao kang	Wang		Doctor	Shandong Provincial Hospital	Jinan, Shandong, China	Principal investigator	The Chinese Multicenter EUGR Collaborative Group			
Ye	Liu		Doctor	Shanghai Children's Medical center	Shanghai, China	Principal investigator	The Chinese Multicenter EUGR Collaborative Group			
Juan	Yi		Doctor	Hunan Children's Hospital	Changsha, Hunan, China	Principal investigator	The Chinese Multicenter EUGR Collaborative Group			
Mei gui	Wu		Doctor	Guangzhou Women and Children's M	Guangzhou, Guangdong, China	Principal investigator	The Chinese Multicenter EUGR Collaborative Group			
Shi feng	Chen		Master	The first people's hospital of yulin	Yvlin, Guangxi, China	Principal investigator	The Chinese Multicenter EUGR Collaborative Group			
Qiao mian	Zhu		Master	Xian Children's Hospital	Xian, Shanxi, China	Principal investigator	The Chinese Multicenter EUGR Collaborative Group			
Lin lin	Wang		Master	Baoji Maternal and Child Health Hosp	Baoji, Shanxi, China	Principal investigator	The Chinese Multicenter EUGR Collaborative Group			
Yong qiao	Liu		Master	People's Hospital of Xinjiang Uygur A	Urumchi, Xinjiang, China	Principal investigator	The Chinese Multicenter EUGR Collaborative Group			

Supplemental Online Content: Nonauthor Collaborators
*First name, last name, and suffix (if applicable) are required and will appear in PubMed.

***First Name and Middle Initial(s)**	***Last Name**	***Suffix (eg, Jr, III)**	Academic Degrees	Institution	Location (city, state/province, country)	Role or Contribution, eg, chair, principal investigator	Group (if more than 1 Group listed in the byline) and/or Subgroup (eg, Steering Committee)			
Chun	Deng		Doctor	Children's Hospital of Chongqing Me	Chongqing, China	Principal investigator	The Chinese Multicenter EUGR Collaborative Group			
Xiao hong	Liu		Master	Affiliated Hospital of Qingdao Univer	Qingdao, Shandong, China	Principal investigator	The Chinese Multicenter EUGR Collaborative Group			
