# Supplementary material for: Antenatal Corticosteroids and Bronchopulmonary Dysplasia in Very Preterm Infants
Source: JAMA Netw Open. 2025 Nov 26;8(11):e2545606. doi: 10.1001/jamanetworkopen.2025.45606 (PMC12658673; doi:10.1001/jamanetworkopen.2025.45606)
Supplement: Supplement 3. — Data Sharing Statement [file jamanetwopen-e2545606-s003.pdf]

Data Sharing Statement

Gao. Antenatal Corticosteroids and Bronchopulmonary Dysplasia in Very Preterm Infants. *JAMA Netw Open*. Published November 26, 2025. doi:10.1001/jamanetworkopen.2025.45606

Data
Data available: Yes
Data types: Deidentified participant data
How to access data: The datasets used during the current study are available from the corresponding author upon reasonable request
When available: With publication

Supporting Documents
Document types: Statistical/analytic code
How to access documents: The R language instruction set used to build the structural equation would been accessed in supplment 4.
When available: With publication

Additional Information
Who can access the data: Researchers whose proposed use of the data has been approved
Types of analyses: Purpose for medicine analyses
Mechanisms of data availability: The data will be made available after approval of a proposal
